# Clinical and Genetic Heterogeneity of HCM: The Possible Role of a Deletion Involving *MYH6* and *MYH7*

**DOI:** 10.3390/genes16020212

**Published:** 2025-02-10

**Authors:** Giancarlo Mancuso, Marina Marsan, Paola Neroni, Consolata Soddu, Francesco Lai, Laura Serventi, Milena Cau, Alessandra Coiana, Federica Incani, Stefania Murru, Salvatore Savasta

**Affiliations:** 1 Medical Genetics Unit, Department of Medical Sciences and Public Health, University of Cagliari, 09124 Cagliari, Italy; giancarlomancuso5@gmail.com (G.M.);; 2 Pediatric and Rare Diseases Clinic, Microcitemico Hospital “A. Cao”, Department of Medical Sciences and Public Health, University of Cagliari, 09124 Cagliari, Italy; salvatore.savasta@unica.it; 3 Neonatal Intensive Care Unit, Department of Surgical Sciences, University of Cagliari, 09124 Cagliari, Italy; 4 Pediatric and Rare Diseases Clinic, Microcitemico Hospital “A. Cao”, ASL 8 Cagliari, 09121 Cagliari, Italy; 5 Unit of Oncology and Molecular Pathology, Department of Biomedical Sciences, University of Cagliari, 09124 Cagliari, Italy; 6 Genetic and Genomic Laboratory, Pediatric Children Hospital “A. Cao”, ASL 8 Cagliari, 09121 Cagliari, Italy; 7 Department of Medical Sciences and Public Health, University of Cagliari, 09124 Cagliari, Italy

**Keywords:** pediatric hypertrophic cardiomyopathy, *MYH7*, *MYH6*, copy number variation

## Abstract

Background/Objectives: Pediatric hypertrophic cardiomyopathy (HCM) is the most common genetic myocardial disorder in children and a leading cause of sudden cardiac death (SCD) among the young. Its phenotypic variability, driven by incomplete penetrance and variable expressivity, presents significant challenges in diagnosis and clinical management. Methods: In this study, we report a unique case of a 16-month-old female diagnosed with HCM caused by a rare genetic deletion. Molecular analysis was performed using a multigene panel and chromosomal microarray analysis (CMA). Results: Molecular tests identified a 30 kb deletion encompassing the *MYH6* and *MYH7* genes. These genes are critical components of sarcomeric architecture, with known associations to HCM and other cardiomyopathies. Conclusions: This case underscores the clinical and genetic heterogeneity of HCM, highlighting the importance of considering genomic deletions involving key sarcomeric genes in the diagnostic evaluation.

## 1. Introduction

Pediatric hypertrophic cardiomyopathy (HCM) represents the most common genetic myocardial disorder in children and a leading cause of sudden cardiac death (SCD) in the young [1]. HCM is characterized by an increased left ventricular thickness, often asymmetrical and involving the interventricular septum, in the absence of other systemic or metabolic causes [2]. Although its prevalence in the general population is estimated at 1 in 500, pediatric cases are far rarer, with incidences ranging between 0.24 and 0.47 per 100,000 children annually [3,4,5]. The clinical manifestation of pediatric HCM is highly variable, ranging from asymptomatic forms to severe heart failure, with onset spanning from infancy to adolescence [6]. The condition is associated with multiple complications, including left ventricular outflow tract obstruction, heart failure, and/or risk of ventricular arrhythmias and sudden cardiac death (SCD) [7]. Phenotypic expression in HCM demonstrates significant variability due to incomplete penetrance and variable expressivity, complicating both risk stratification and clinical management [8]. In the last 30 years, after the genetic cause of HCM was first recognized, over 1000 mutations have been identified in various genes, with the most common involving eight sarcomere protein-encoding genes: *MYH7*, *MYBPC3*, *TNNT2*, *TNNI3*, *TPM1*, *MYL2*, *MYL3,* and *ACTC1*. *MYH7* and *MYBPC3* account for up to 75% of identified mutations, while the others each contribute 1–5% of cases. Rare variants in additional genes associated with the sarcomere apparatus, Z-disc, or calcium homeostasis pathways (e.g., *MYH6*, *TTN*, *PLN*) have also been identified, some of which may act as modifiers rather than direct pathogenic factors [9]. HCM in children differs markedly from adult forms in terms of etiology, clinical course, and management. While mutations involving sarcomeric genes, including *MYH7* and *MYBPC3*, are the leading cause in both populations, pediatric cases exhibit greater etiological heterogeneity [10]. In particular, *MYH7* is responsible for a form of cardiomyopathy, hypertrophic, 1 (OMIM: #192600). Advances in genetic testing, including next-generation sequencing (NGS), have transformed the diagnostic landscape of HCM, enabling the identification of pathogenic variants in 30–60% of cases [11,12]. Several genes have been identified in the past years as possible candidate genes in association with HCM [13,14]. Among these, the *MYH6* gene has been associated with a spectrum of cardiomyopathy phenotypes, encompassing hypertrophic cardiomyopathy (OMIM: #613251), including severe cases with early onset and subsequent progression to ventricular dilation, as well as dilated cardiomyopathy (DCM), characterized by ventricular dysfunction and a gradual disease course [15]. In recent years, pathogenetic variants on the *MYH6* gene have also been correlated with atrial septal defects [16,17] and hypoplastic left heart syndrome [18]. The *MYH6* gene is located on the long arm of chromosome 14, and it belongs to the family of sarcomeric protein genes [19]. It encodes α-myosin heavy chain (α-MHC), a critical motor protein for cardiac muscle contraction, predominantly expressed in the atria throughout postnatal life [18]. During early cardiac development, *MYH6* is also expressed in the ventricles but becomes largely replaced by *MYH7*, which encodes β-myosin heavy chain (β-MHC), in postnatal ventricular tissue. Despite its low expression in non-failing ventricles, *MYH6* pathogenetic variants have been implicated in a spectrum of cardiomyopathies, suggesting a role in ventricular function under pathological conditions [20]. In this study, we discuss the involvement of *MYH6* and *MYH7* genes in HCM, focusing on the possible pathological role of a large gene deletion.

## 2. Materials and Methods

### 2.1. Sample Collection, Genomic DNA Extraction and Library Preparation

Written informed consent for blood sample collection and genetic analysis was obtained from the proband and their parents in compliance with the Declaration of Helsinki. Genomic DNA was extracted from peripheral blood samples using the QIAamp^®^ DNA Blood Kit (Qiagen, Hilden, Germany) following the manufacturer’s protocol.

A custom Ion AmpliSeq panel was designed using the Ion AmpliSeq Designer v7.4.8.3 tool (Ion Torrent Systems Inc., Gilford, NH, USA). For the sequencing procedure, libraries were prepared by amplifying 10 ng of genomic DNA from each proband using the Ion AmpliSeq Library Kit v2.0 (Ion Torrent Systems Inc., Waltham, MA, USA), according to the manufacturer’s instructions. Sample multiplexing was achieved by barcoding each library with the Ion Xpress Barcode Adapters (Ion Torrent Systems Inc., Waltham, MA, USA). According to the manufacturer’s instructions, the libraries were loaded and subsequently sequenced on the Ion Chef-PGM system Ion Torrent Systems Inc., Waltham, MA, USA). Raw sequencing data were analyzed using Ion Torrent Suite™ Software v5.12.1, including Variant Caller v5.12.0.4 and Coverage Analysis v5.12.0.0. The analysis workflow involved base calling, quality control of raw data, sequence alignment against the reference human genome (hg19), and variant calling. The resulting aligned reads (BAM files) were further processed and annotated using Ion Reporter™ Software v5.20 (Ion Torrent Systems Inc., Waltham, MA, USA).

### 2.2. Variant Calling and Prioritization

The identified variants were filtered by selecting only the coding regions and intron-exon junctions of the analyzed genes. Synonymous variants and variants reported in population databases with a frequency greater than 1% were excluded from the survey. Variants were selected and classified based on the following criteria: (1) consistency with the clinical suspicion and expected inheritance pattern; (2) presence of the variant in pathology databases and scientific literature; (3) assessment of the degree of evolutionary conservation; and (4) prediction of the variant’s pathogenicity. The clinical significance of the variants was evaluated in accordance with the 2015 guidelines issued by the American College of Medical Genetics and Genomics (ACMG). The reference databases used for the classification of variants were ClinVar and HGMD Professional.

### 2.3. Single Nucleotide Polymorphism (SNP) Array

It was performed using the Infinium CytoSNP 850 K v1.3 microarray, spanning the entire genome, with a resolution of 100 Kb, according to the instructions provided by the manufacturer. The Illumina Infinium CytoSNP 850K v1.3 Bead Chip platform and scanning using NextSeq550 was used for SNP-array analysis. The level of resolution, dependent on the number of SNPs and their distance in the investigated region, is between 5 and 1 Kb in the regions, including relevant genes as indicated by ICCG (International Collaboration for Clinical Genomics) and CCMC (Cancer Cytogenomics Microarray Consortium). The analysis of CNVs (Copy Number Variants) and regions with LOH (loss of heterozygosity) is performed using Bluefuse Multi Software v4.3. Map positions refer to the Human Genome Reference Consortium (hg19). CNVs of loci known to be polymorphic (benign nonpathogenic sites reported in the Available online: https://dgv.tcag.ca/dgv/app/home (10 October 2024)) or considered likely benign variants, or regions with LOH sizes less than 8/10 Mb, were not included in the results. The analysis is unable to detect balanced chromosomal rearrangements, unbalanced rearrangements in pericentromeric or pseudoautosomal X/Y regions, and low percentage mosaicisms (<20%).

## 3. Results

### Case Presentation

**Figure 1:**
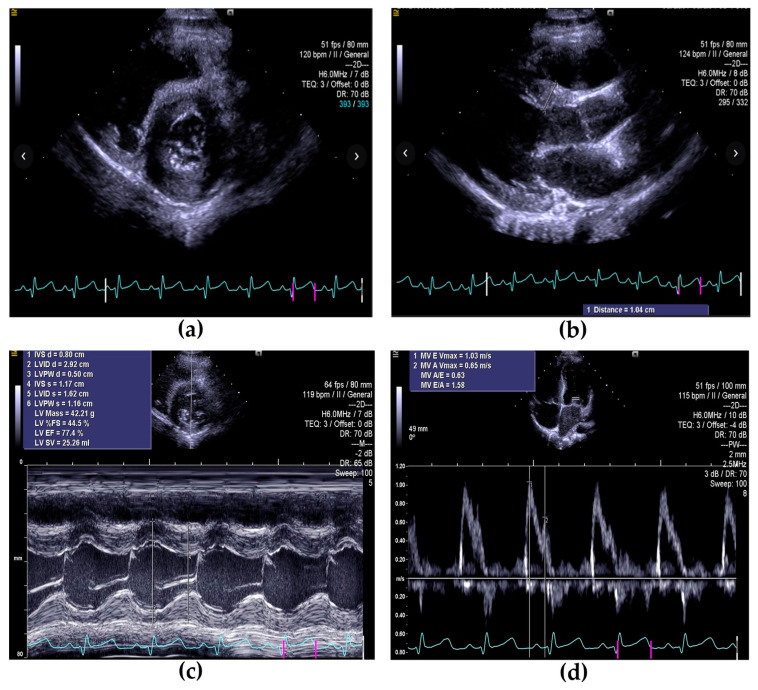
Transthoracic echocardiography showing hypertrophic cardiomyopathy: (**a**) Parasternal short axis view shows hypertrophy of the ventricle; (**b**) parasternal long axis view shows increased thickness of the interventricular septum; (**c**) parasternal short axis view M-Mode, shows thickened posterior ventricular wall; (**d**) apical four-chamber view shows transmitral pattern E/A equal to 1.58.

A 16-month-old female toddler was referred to our Pediatric Rare Diseases Department for a genetic assessment prompted by ultrasound findings of a pulmonary valve stenosis and non-obstructive ventricular hypertrophy. She was born premature at 34 weeks and 4 days of gestation by a cesarean section at the end of a physiological pregnancy. Serial gynecological ultrasounds throughout gestation revealed no abnormalities, and first-trimester biochemical exams were within limits. APGAR score at 1 min was 7, at 5 min was 9, and at 10 min was 10. During hospitalization, she underwent physical examination, blood exams, abdominal and cerebral ultrasounds, and auditory and ophthalmic screening exams, all of which were reported to be within limits. The echocardiogram and cardiac examination showed a patent ductus arteriosus (PDA) with no hemodynamic relevance, and for which she started a cardiological follow-up program to be held every 6 months. At 15 months of age, the echocardiogram revealed augmented ventricular walls (interventricular septum Z-score was +2.4; posterior left ventricular wall Z-score was +1.2; LVDD Z-score was −2; LVSD Z-score was −2), as shown in Figure 1, and a mild pulmonary valve stenosis (peak transpulmonary gradient: 25 mmHg), and she was therefore sent to our department on the basis of a suspected genetic condition.

**Figure 2:**
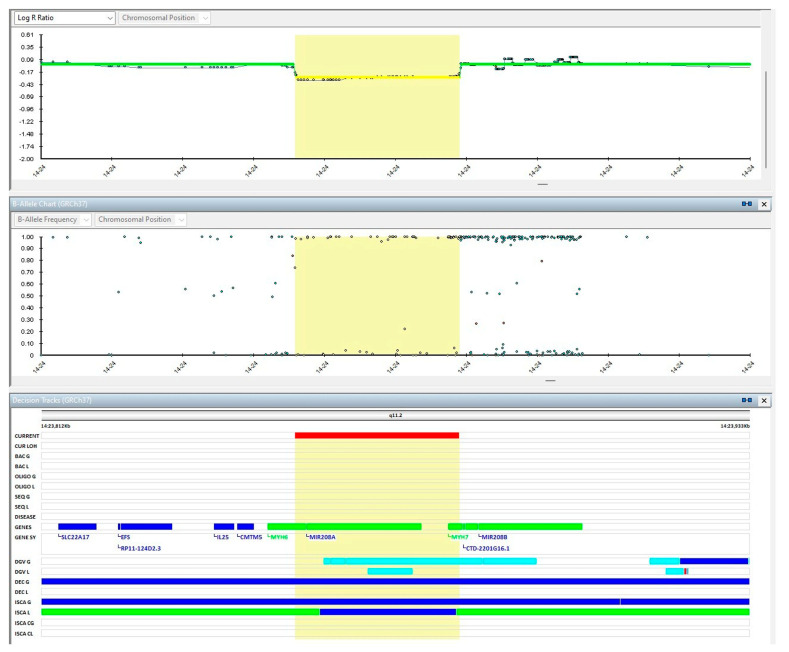
SNP-array showing the deletion involving *MYH6*-*MYH7* genes in the proband. The yellow banner shows the range of the deletion, from chr14: 23,386,269 to 23,416,678. Map positions refer to the Human Genome Reference Consortium (hg38).

A thorough medical history was collected, which disclosed the presence of a tricuspid insufficiency in the father. No other major diseases or cardiovascular abnormalities were reported in the patient’s family across three generations. No environmental factors that might contribute to disease onset were referred to or detected. Her physical examination exhibited minor dysmorphic features, such as an epicanthal fold on the right, a short neck, diastasis recti, and umbilical hernia; she also had a systolic murmur of 2/6. Nonetheless, the cardiac abnormalities and their onset initially prompted the execution of a multi-gene panel of 17 genes related to RASopathies, but no pathogenetic variants were found. Due to hypertrophic cardiomyopathy, the DNA sample of the proband was analyzed by NGS using a panel of genes classically associated with HCM. The NGS analysis identified the heterozygous deletion of exons from 38 to 40 of the *MYH7* gene. To better define the extension of the deletion underlined by the multi-gene panel, a chromosomal microarray analysis (CMA) through single nucleotide polymorphism (SNP) Array was performed. The SNP-array exam showed the presence of a deletion of 30 Kb involving the long arm of chromosome 14 and including the *MYH7* gene (from exon 34 to exon 40) and the *MYH6* gene (from exon 1 to exon 33). Due to the identification of this deletion, a segregation study of the family was performed, which revealed the paternal inheritance of the deletion. The echocardiographic evaluation did not show any sign of cardiomyopathy in the siblings (Figure 2). Currently, the affected patient undergoes follow-up evaluations every six months, while the other family members are monitored annually. During these routine clinical assessments, the patient’s condition has remained stable, with no need for pharmacological or surgical interventions. Potential pharmacological treatments will be considered based on future clinical developments and the patient’s condition.

## 4. Discussion

Hypertrophic cardiomyopathy is a genetic heart condition characterized by an increased left ventricular wall thickness in the absence of systemic disorders or secondary causes that can lead to left ventricular hypertrophy (LVH) [21,22] and is a leading cause of sudden cardiac death in adolescents and young adults [1]. It is usually inherited with an autosomal dominant pattern and variable penetrance and expression [2]. Thanks to SCD risk stratification and cardiovascular therapies and interventions [23], HCM mortality rates have lowered to <1% a year [24], with heart failure being the main cause. Other causes may be malignant ventricular arrhythmias, myocardial ischemia, and diastolic dysfunction [25,26]. All the above can be explained by the anatomical alterations that characterize HCM, such as left ventricular outflow obstruction (LVOTO), diastolic dysfunction, mitral regurgitation, myocardial ischemia, arrhythmias, and autonomic dysfunction [22,27,28,29]. Generally, the diagnostic work up for HCM is prompted by a positive family history of HCM, occurrence of symptoms, or abnormalities found in routine screening EKG or echocardiography [22]. Firstly, all causes of secondary LVH must be excluded: these include syndromes such as RASopathies, mitochondrial myopathies, metabolic diseases, and all conditions that may lead to remodeling of the ventricular wall, like chronic arterial hypertension or hemodynamic obstructions of the left side of the heart [22]. HCM is then established through noninvasive cardiac imaging, including echocardiography and/or cardiac MRI, and genetic testing, which either usually identifies a sarcomere-related variant or remains inconclusive [25]. In children, left ventricular wall thickness is measured through cardiac ultrasound and then adjusted for age and body surface area: HCM is identified by a Z-score > 2.5 SD in asymptomatic children with no family history, or Z-score > 2 SD in case of positive family history or positive genetic test [22,25]. Although asymmetric septal hypertrophy is the most common finding, the location and degree of LVH may vary due to both the variable phenotypic expression among individuals and also to the fact that the latter may not be fully completed until adolescence or even after [30,31]. For instance, the increased thickness can also present in a concentric pattern of either the entire wall or just the apex. Moreover, obstruction of the left ventricular flow, associated with systolic anterior motion of the mitral valve, mid-ventricular obstruction, or diastolic dysfunction, may also be found in these patients. Genetic counseling is then offered to all patients with unexplained LVH. Usually, a first-line diagnostic gene panel for HCM is performed, which includes the most common sarcomere genes associated with the disease (*MYH7*, *MYBPC3*, *TNNI3*, *TNNT2*, *TPM1*, *MYL2*, *MYL3*, and *ACTC1*) [32], as well as other non-sarcomeric variants with strong evidence of pathogenicity (*CSRP3*, *JPH2*, *ALPK3*, and *FHOD3*). Larger panels may lead to misinterpretation and therefore are not used in the initial tests. Nonetheless, other specific genes may be investigated in case of suspicion of a systemic disorder such as glycogen storage disease (*PRKAG2*), Danon disease (*LAMP2*), Fabry disease (*GLA*), transthyretin amyloid cardiomyopathy (*TTR*), and Pompe disease (*GAA*), for which an early diagnosis could modify therapeutic options. This would not apply in the case of a sarcomeric or non-sarcomeric HCM, as the intra- and inter-familial heterogeneity of HCM does not influence the clinical decision making in terms of therapeutic choices [22]. However, an early diagnosis would help identify patients at risk and allow clinicians to offer them periodic follow-up in order to prevent complications.

In the human cardiac muscle, two main myosin heavy chain (MHC) isoforms are expressed: α-MHC, primarily in the atrial tissue, and β-MHC, dominant in the ventricular tissue. Both isoforms are encoded by tandemly arranged genes, *MYH6* and *MYH7*, located on chromosome 14q12. During postnatal life, the mechanical and hormonal stimuli on cardiac tissue result in a change in the expression of MCH isoforms from fetal to adult, highlighting the adaptability of cardiac muscle [20,33]. Specifically, during the fetal period, the *MYH6* gene encodes for the α-MHC form, which is initially the most highly expressed isoform in the ventricle. Eventually, the α-MHC isoform is replaced by the β-MHC isoform, encoded by the gene *MYH7*, making it the most highly expressed isoform in the human ventricle in the adult period [34,35]. This change between pre- and postnatal life is regulated by different factors, among which there are epigenetic modifications, such as CpG methylation, that play a crucial role in the regulation of cardiomyocyte-specific genes, with a progressive demethylation of CpG regions within these genes [36,37]. Due to the importance of the β-MHC isoform, pathogenic variants of the *MYH7* gene are a major contributor to hypertrophic cardiomyopathy and are often clustered in the converter domain, a critical region for mechanical force transduction [34,35]. Nonetheless, during postnatal life, various stressors in contractile proteins can induce a regression to a fetal-like gene expression profile [37,38,39]. Although the α-myosin heavy chain, encoded by *MYH6*, is predominantly expressed during fetal development and becomes less prominent postnatally, mutations in *MYH6* have been strongly implicated in a range of cardiac diseases, including hypertrophic cardiomyopathy, dilated cardiomyopathy, and congenital heart defects such as atrial septal defects. Such phenotypic variability reflects the gene’s complex influence on sarcomeric architecture and cardiac contractility [40]. These insights reflect the dynamic epigenetic remodeling that underpins cardiomyocyte development and functional specialization throughout life.

Specifically, *MYH6* mutations have been linked to both hypertrophic and dilated cardiomyopathies, with distinct cases exhibiting a transition from a hypercontractile state to myocardial thinning and ventricular dilation. The pathological mechanisms linked to deleterious *MYH7* and *MYH6* variants have been progressively uncovered, though they remain incompletely understood. Studies have demonstrated that the disruption of the *MYH7*-C3 super-enhancer, which plays a pivotal role in the regulatory switch between *MYH6* and *MYH7* during cardiac development, could contribute to the pathogenetic mechanisms of HCM through epigenetic and transcriptional dysregulation. Deletion of the *MYH7*-C3 region disrupts this regulation, resulting in sustained expression of *MYH6* and reduced expression of *MYH7*. This alteration in the α-MHC/β-MHC ratio predisposes the heart to hypertrophic remodeling and functional impairment under stress. The *MYH7*-C2 enhancer, which overlaps the *MYH6* promoter, demonstrated moderate enhancer activity, reflecting its role in regulating *MYH6* [41]. Another proposed mechanism involves the altered composition of sarcomeric proteins. *MYH7* encodes β-MHC, which has slower ATPase activity and is optimized for sustained contraction in adult ventricles, whereas *MYH6* encodes α-MHC, which exhibits faster ATPase activity but higher energy consumption. The deletion likely disrupts the balance between these isoforms, leading to energy inefficiency and increased mechanical stress on the myocardium—both of which are hallmarks of HCM pathogenesis [42]. Furthermore, the deletion-induced imbalance in α-MHC and β-MHC expression may activate compensatory hypertrophic signaling pathways, including the MAPK, PI3K-Akt, and Ca^2+^-dependent pathways. These signaling cascades, triggered by altered biomechanical forces or energy deficits, promote cellular hypertrophy, fibrosis, and apoptosis, thereby exacerbating the clinical phenotype of HCM [43]. Several studies have investigated the role of deleterious variants on *MYH6* and *MYH7* genes in mice, showing how these variants determine an altered function of the gene that may lead to a pathological phenotype [44,45]. To our knowledge, no other reported deletion involving both *MYH6* and *MYH7* genes has been directly associated with HCM in scientific literature. Segregation studies on dominant *MYH6* and *MYH7* mutations linked to congenital heart defects (CHDs) such as atrial septal defects have demonstrated variable penetrance, with several mutation carriers exhibiting no structural heart abnormalities [16,17,46]. Similarly, our study documents variable intra-familial expression, as the proband’s father, carrying the same deletion, remains asymptomatic for HCM. According to current guidelines [31], and after excluding RASopathies, no additional molecular tests were performed in the absence of clinical signs suggesting more complex phenotypes. Several scientific works have investigated the clinical relevance of CNVs in HCM, but no cases similar to ours have been reported in these studies [15,47]. Notably, a recent report described a 5-month-old boy with an atrial septal defect and a deletion encompassing *MYH6* exons 1–26 and *MYH7* exons 28–40. This case highlights the potential pathogenic significance of such deletions, suggesting they may contribute to the development of cardiac phenotypes [48]. Furthermore, three similar deletions were identified on Decipher (available online: https://www.deciphergenomics.org/ (27 January 2025)). One of these, patient 289066, exhibited a comparable deletion and a diagnosis of cardiomyopathy. For the other two patients, no cardiological data was available. These findings emphasize the critical need to investigate the role of combined deletions in *MYH6* and *MYH7* in the context of HCM and related disorders. A major challenge in the field remains the incomplete understanding of how genetic variations in sarcomeric genes contribute to HCM pathogenesis. Comprehensive genetic testing is essential for the early identification of at-risk individuals, enabling timely intervention before clinical complications occur. Insights into the molecular mechanisms underlying HCM will lead to advances in treatment strategies, facilitate preventive measures, and enhance genetic counseling for affected families. We further highlight the necessity for ongoing research into the functional consequences of *MYH6* and *MYH7* deletions to improve our understanding of their role in the spectrum of cardiac diseases.

## 5. Conclusions

Our study contributes to understanding the genotype–phenotype correlation in pediatric HCM, emphasizing the potential significance of deletions involving causative genes such as *MYH6* and *MYH7*. Our study has some limitations, particularly the absence of cardiac tissue samples, which prevented us from conducting functional studies to directly assess the physiological consequences of the identified deletion on myocardial function. The inability to perform these studies leaves uncertainty regarding the exact impact of the deletion on cardiac performance. Additionally, as this is a single case report, further studies involving larger cohorts or additional cases are necessary to validate and strengthen the proposed genotype–phenotype correlation. Further studies with in vitro and in vivo models focusing on deletion involving these critical genes may help to clarify their role in cardiac pathophysiology.

## Data Availability

The original contributions presented in this study are included in the article. Further inquiries can be directed to the corresponding author.

## Abbreviations

The following abbreviations are used in this manuscript:

HCM	Hypertrophic cardiomyopathy
SCD	Sudden cardiac death
CMA	Chromosomal microarray analysis
NGS	Next-generation sequencing
DCM	Dilated cardiomyopathy
α-MHC	α-myosin heavy chain
β-MHC	β-myosin heavy chain
ACMG	American College of Medical Genetics and Genomics
SNP	Single nucleotide polymorphism
ICCG	International Collaboration for Clinical Genomics
CCMC	Cancer Cytogenomics Microarray Consortium
CNVs	Copy number variants
LOH	Loss of heterozygosity
PDA	Patent ductus arteriosus
LVH	Left ventricular hypertrophy
LVOTO	Left ventricular outflow obstruction
MHC	Myosin heavy chain
CHDs	Congenital heart defects
